# Modelling structural properties of cyanine dye nanotubes at coarse-grained level†

**DOI:** 10.1039/d2na00158f

**Published:** 2022-06-20

**Authors:** Ilias Patmanidis, Paulo C. T. Souza, Selim Sami, Remco W. A. Havenith, Alex H. de Vries, Siewert J. Marrink

**Affiliations:** Groningen Biomolecular Science and Biotechnology Institute, University of Groningen Nijenborgh 7 Groningen 9747 AG the Netherlands s.j.marrink@rug.nl; Zernike Institute for Advanced Materials, University of Groningen Nijenborgh 4 Groningen 9747 AG The Netherlands; Molecular Microbiology and Structural Biochemistry, UMR 5086 CNRS and University of Lyon Lyon France; Stratingh Institute for Chemistry, University of Groningen Nijenborgh 4 Groningen 9747 AG The Netherlands; Ghent Quantum Chemistry Group, Department of Chemistry, Ghent University Krijgslaan 281 (S3) B-9000 Gent Belgium

## Abstract

Self-assembly is a ubiquitous process spanning from biomolecular aggregates to nanomaterials. Even though the resulting aggregates can be studied through experimental techniques, the dynamic pathways of the process and the molecular details of the final structures are not necessarily easy to resolve. Consequently, rational design of self-assembling aggregates and their properties remains extremely challenging. At the same time, modelling the self-assembly with computational methods is not trivial, because its spatio-temporal scales are usually beyond the limits of all-atom based simulations. The use of coarse-grained (CG) models can alleviate this limitation, but usually suffers from the lack of optimised parameters for the molecular constituents. In this work, we describe the procedure of parametrizing a CG Martini model for a cyanine dye (C8S3) that self-assembles into hollow double-walled nanotubes. First, we optimised the model based on quantum mechanics calculations and all-atom reference simulations, in combination with available experimental data. Then, we conducted random self-assembly simulations, and the performance of our model was tested on preformed assemblies. Our simulations provide information on the time-dependent local arrangement of this cyanine dye, when aggregates are being formed. Furthermore, we provide guidelines for designing and optimising parameters for similar self-assembling nanomaterials.

## Abstract 2

Coarse-grained models are essential to capture the self-assembly of supramolecular materials.

## Graphical abstract

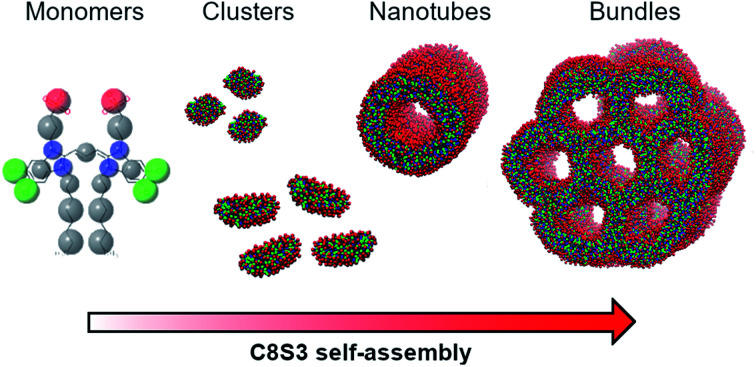

Self-assembly describes the process of small building blocks organising into large supramolecular structures.^1^ Under certain conditions, self-assembly occurs spontaneously driving the systems towards favourable states in terms of free energy. The chemical nature and structure of the building blocks, together with the solvent properties and state conditions, determine the aggregation pathways and the type of the final aggregate. Non-covalent interactions are dominant in this process, and they play a crucial role in determining the properties and functionality of the self-assembled structures.

Cyanine dyes are a family of molecules whose members are known to aggregate and form different types of structures with discrete features.^2,3^ A typical structure of a cyanine dye contains a polymethine bridge, a chain with an odd number of methine groups (=CH–) connected *via* alternating single and double bonds, with nitrogen atoms on both ends (see Fig. 1a). The conjugated system created by the pi-orbitals of the polymethine bridge provides a chromophore responsible for the optical signature of each cyanine dye. In different solvents, cyanine dyes form organised structures that exhibit different optical properties compared to the solvated monomers. Such special optical properties were first reported for the pseudo-isocyanine (PIC) dye.^4,5^ Later, it was suggested that the strong coupling of the individual molecules was responsible for the spectral changes, which depend on the structure of the aggregates and the relative orientation of the monomers.^6,7^ Understanding the driving forces that govern the formation of specific structures and the monomer arrangement inside the aggregates is of major importance in order to design molecules with specific chemical features and, ultimately, predict their supramolecular structures and properties. Computational modelling, in particular molecular dynamics (MD) simulations, are a powerful tool to aid in the rational design process of supramolecular biomolecules and nanomaterials.^8,9^

**Fig. 1:**
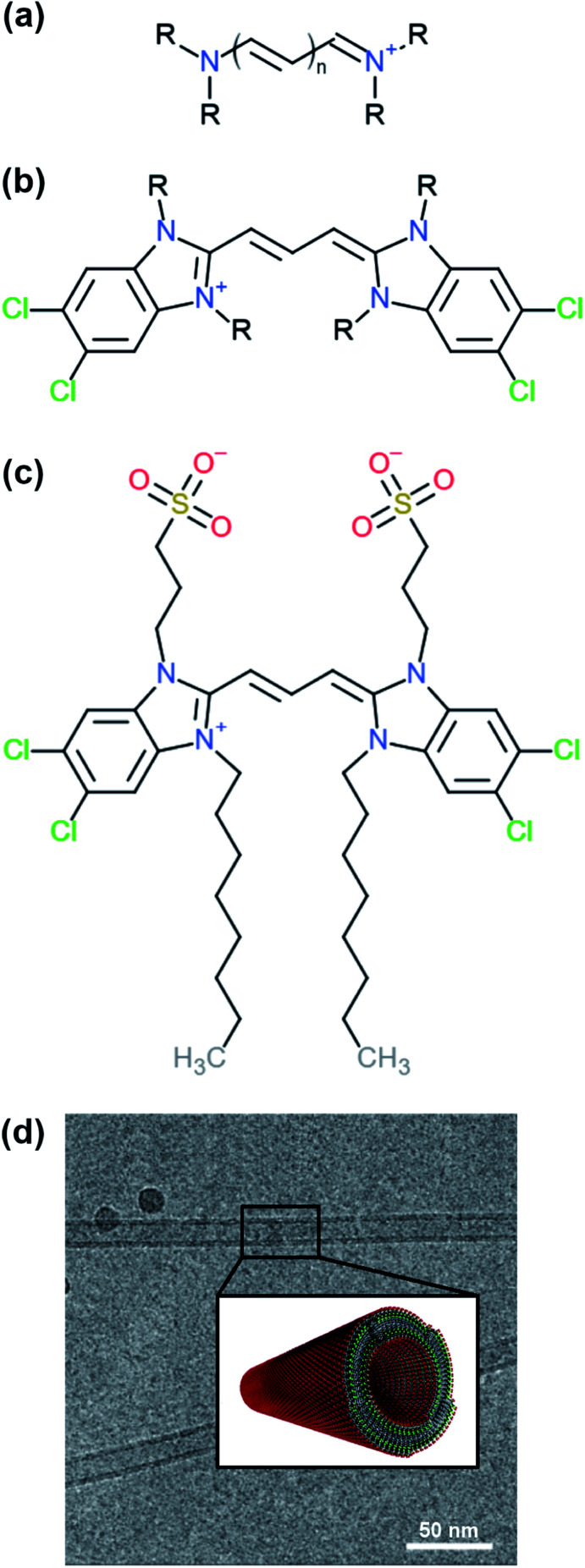
Examples of different cyanine dyes. (a) Generic cyanine dye chemical type, (b) 5,5′,6,6′-tetrachloro-benzimida-carbocyanine. The R-tails can be substituted with various chemical groups giving rise to different types of aggregates. (c) Chemical structure of a C8S3 molecule. (d) Structure of C8S3 nanotubes obtained from electron microscopy experiments and a graphical representation of a nanotube.^10^ The image was obtained and adapted from the original publication.

One of the cyanine dyes that has been extensively studied the past decades is 5,5′,6,6′-tetrachloro-benzimida-carbocyanine^11,12^ (see Fig. 1b). Several molecules with this specific core and different substituents have been designed and synthesised, and their structural and optical properties have been examined.^13,14^ Interestingly, different types of aggregates were formed ranging from bilayers and ribbons, to wires and nanotubes, depending on the chemical nature of the substituents and the experimental conditions. In this study, we were interested in 1,1′-dioctyl-3,3′-bis(2-sulfopropyl)-5,5′,6,6′-tetrachloro-benzimida-carbocyanine (C8S3) (see Fig. 1c), which forms double-walled nanotubes^14–18^ (see Fig. 1d). C8S3 aggregates have been previously studied with MD simulations by testing the stability of different types of aggregates and their optical properties.^19–21^ These studies were limited to simulations of preformed aggregates, that do not necessarily allow the molecules to rearrange and explore a large portion of the available configurational space.

Intrinsic pitfalls of atomistic MD simulations render the task of obtaining ordered aggregates from self-assembly simulations extremely difficult or even beyond the realm of feasibility, at least by using brute force atomistic MD simulations.^8^ The main issues are the time scales, at which self-assembly occurs, and the free energy landscape of the procedure, which is rugged with many local minima in which the systems get trapped. Coarse-grained (CG) approaches are a common solution to alleviate these problems. The simplified representation of the atomic coordinates and the smaller number of interactions that need to be calculated in CG simulations offer significant speed-ups and smoothen the free energy landscape, facilitating the formation of self-assembled structures.^22–25^

In this work, we designed a CG model able to represent the spatial and dynamic properties of the C8S3 cyanine dye. To achieve this, we optimised the atomistic parameters for the aromatic core based on quantum mechanics (QM) calculations and tested their performance on available crystal structures of cyanine dyes. The optimised atomistic parameters were used as a reference for tuning the bonded parameters of a CG model with the latest version of the Martini force field, Martini 3.^26^ Its fastidious parametrization procedure, the flexible mapping scheme and the transferability of the building block approach underlying Martini^27^ allowed us to construct an accurate representation of the cyanine dye aromatic core and its side chains. The final CG model was tested in C8S3 self-assembly and preformed nanotube simulations. Our results provide insights on the local and global arrangement of C8S3 molecules inside the aggregates. Furthermore, they provide a template for the aromatic core of similar cyanine dyes, since the same core could be used to study other members of the cyanine dye family. The workflow for constructing similar CG models is described in detail.

## 1 Methods

### 1.1 Optimising atomistic parameters

**Fig. 2:**
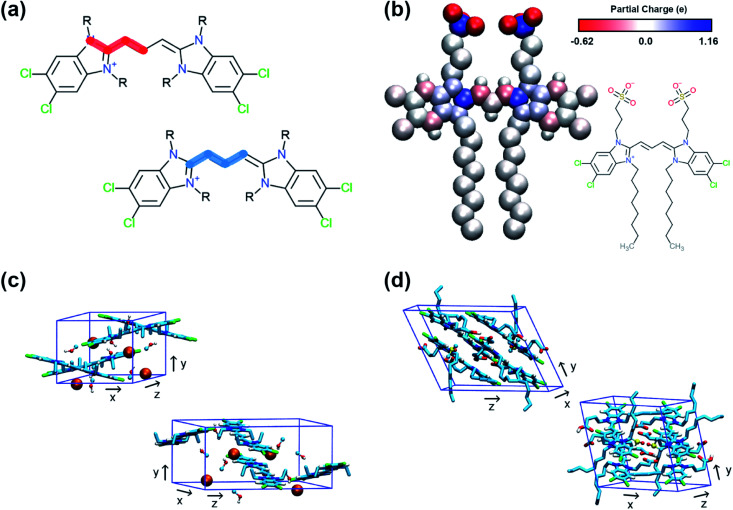
Details of the atomistic models' parametrization. (a) Dihedral angles of the aromatic core that were optimised with Q-force.^28^ (b) Partial charges of C8S3 molecule derived from QM. (c) Unit cell of C2C2 crystals. (d) Unit cell of C8O3 crystals.

QM methods were used to obtain different sets of parameters, whose performance was evaluated in atomistic simulations. Specifically, the atomistic model was optimised in terms of bonded potentials of the aromatic core linker and partial charges (see Fig. 2a and b). Optimisation was required in the dihedral angles of the polymethine bridge to ensure that the large conjugated system of the cyanine dyes is adequately described and can be used for different cyanine dyes. The torsional potentials that describe the dihedral angles of the linker have been determined based on the energy difference between the QM and the MD dihedral profiles using Q-force.^28^ For the partial charges, the Dipole Preserving Analysis (DPA)^29^ using the GAMESS-UK software,^30^ and the Restricted Electrostatic Potential (RESP)^31^ method using the Gaussian16 software^32^ were employed to obtain different sets of parameters and test their performance. The procedure for obtaining parameters and the final comparison are included in Table S1–S3 and Fig. S1 of the ESI.†

### 1.2 Crystal structures of cyanine dyes

The crystal structures of two cyanine dyes with the same aromatic core as C8S3, but different substituents (namely C2C2 (ref. 33) and C8O3 (ref. 34)) were simulated to evaluate the performance of the QM generated parameters. The unit cell of each crystal is presented in Fig. 2c and d. To prepare the initial structure for the simulations, the unit cells for C2C2 and C8O3 were replicated along each dimension by a factor of 3, resulting in supercells consisting of 27 unit cells and 108 cyanine dye molecules. C2C2 crystal structures also included 108 iodine molecules to neutralise the charge and 216 methanol molecules, whereas C8O3 crystal structures included 54 dimethyl-sulfoxide (DMSO) molecules and no counter ions. Our goal was to find the best set of parameters that is able to maintain the experimental determined structures and, then, use it as reference for the CG model. The atomistic parameters were evaluated based on their ability to maintain structural features of the initial unit cells, such as box dimensions and density. The results from the crystal simulations are reported in Table S4 of the ESI.†

### 1.3 MD simulations – atomistic

All simulations were performed with the GROMACS 2018 simulation package.^35,36^ For the atomistic simulations, an extended version of the GROMOS force field version G54a7 (ref. 37) was used that incorporates recent parameters for molecules from the ATB database^38^ and can be found on the ATB website (https://atb.uq.edu.au/). In this version, there is an additional atom type for carbons in aromatic rings (CAro) that was used for representing the carbons of the cyanine core. Furthermore, parameters for iodine (ATB Molecule ID: 337757), DMSO (ATB Molecule ID: 1202) and methanol (ATB Molecule ID: 15607), that were present in the crystal structures of the cyanine dyes, were downloaded from the ATB database. Simulations with solvated cyanine dyes in water were used to obtain distributions for the bonded terms of the CG model. In those cases, explicit SPC water molecules^39^ were used to model water, and counter ions (Na^+^) were introduced to neutralise the systems.

Regarding pressure coupling, the Berendsen barostat^40^ was used to maintain the pressure constant at 1 bar with a time constant of 1 ps and compressibility of 4.5 × 10^−5^ bar^−1^. Isotropic pressure coupling was used for most systems. On the contrary, the crystal simulations were performed in anisotropic boxes, where the pressure of each dimension of the box was decoupled from the other dimensions. In all systems, the temperature was kept constant at 300 K by using the *v*-rescale algorithm with a time constant of 0.1 ps.^41^ The cut-off for electrostatic and van der Waals interactions was set to 1.4 nm and the Verlet update scheme^42^ was used for the short range non-bonded interactions with a buffer tolerance of 0.005 kJ mol^−1^ ps^−1^. Long range interactions were calculated with the Reaction Field method^43^ for maximum performance during the production phase. The LINCS algorithm was employed for constraining the bond lengths.^44^

All systems were minimised for 1000 steps using the Steepest Descent algorithm. The production phase of the crystal simulations started immediately after the minimisation and it lasted 200 ns. In contrast, the systems with solvated cyanine dyes in water were equilibrated first in NVT and then in NPT conditions, each for 10 ps with a 1 fs time step. Their production phase was performed in the NPT ensemble and its duration was 100 ns. The time step for integration was set to 2 fs for the production phase of all atomistic simulations.

### 1.4 MD simulations – CG

For all CG simulations, the Martini 3 force field^26^ was used. C8S3 monomers and aggregates were solvated in Martini 3 water beads (W, representing 4 H_2_O molecules), and Na^+^ (TQ5 bead with +1 charge) were used to neutralise the charge of each system. In all systems, the Berendsen barostat^40^ was used to maintain the pressure constant at 1 bar with a time constant of 1 ps and compressibility of 3 × 10^−4^ bar^−1^. The temperature was kept constant at 300 K by using the v-rescale algorithm with a time constant of 0.1 ps.^41^ Electrostatic and van der Waals interactions were calculated using the Verlet list scheme^42^ with the cut-off set at 1.1 nm and a buffer tolerance of 0.01 kJ mol^−1^ ps^−1^. Long range interactions were treated using the Reaction Field method^43^ and the bond lengths were constrained by using the LINCS method.^44^

Regarding the self-assembly simulations, each system was minimised for 1000 steps using the Steepest Descent algorithm. Then, a short equilibration step followed in the NPT ensemble for 100 ps with a time step of 10 fs. The production phase for these systems was 10 μs and the time step of integration was 20 fs.

Furthermore, nanotubes and a nanotube bundle with length ∼50 nm (∼3650–6000 and ∼16 000 molecules, respectively) were constructed and solvated in rectangular boxes. The C8S3 nanotubes were constructed by creating 2D-lattices from an initial unit cell and, then, rolling the lattices into cylindrical shapes.^20,21^ The initial unit cell was obtained from the crystal structure of a cyanine dye with the same aromatic core as the C8S3 molecule,^33^ and the original molecules were replaced with C8S3 molecules at CG level. Using the same protocol, a nanotube bundle was designed by placing C8S3 nanotubes in close distance (∼9 nm between the centre of each nanotube) and removing the parts of the outer wall that were within the area of the bundle and at the cross section of each nanotube. Details for the arrangement and the dimensions of the nanotubes are reported in Table S6 of the ESI.†

The systems were minimised for 1000 steps and equilibrated in the NPT ensemble for 1 ns with a time step of 10 fs. During equilibration, harmonic potentials were used to restrain the aromatic core and allow the tails to relax. Their force constant was 100 kJ mol^−1^. Despite the presence of the restraints, the large number of concentrated charged beads in the hydrophilic tails created instabilities in the inner and outer wall of the nanotubes that resulted in small holes. To ensure that the nanotube remained intact during the equilibration phase, the equilibration process was performed in three steps, and the charges of the beads were progressively increased to their normal value (−0.1, −0.5 and −1.0). The actual nanotube simulations lasted 1 μs, and the time step during the production phase was 20 fs.

## 2 Results and discussion

### 2.1 CG model preparation

In this section, the structural and chemical properties of the C8S3 molecule will be discussed, and how these properties were tuned to create an accurate CG model. Specifically, we will focus on mapping, bead choices, surface area and the bonded distributions. The next paragraphs offer general guidelines on choosing and improving the topologies of similar molecules with Martini.

#### 2.1.1. Mapping

**Fig. 3:**
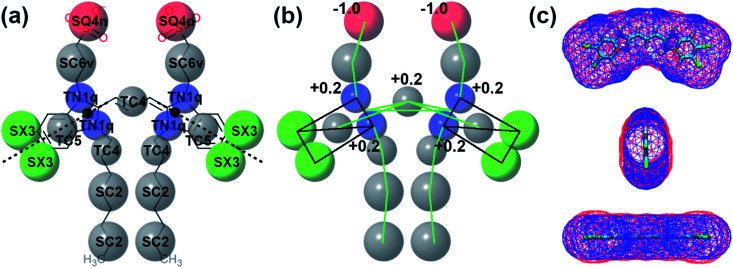
Details of C8S3 CG model. (a) Mapping and bead types. The bead types of CG model are superimposed to its chemical structure. Each bead is colored based on its type (C: grey, N: blue, X: green and Q: red). The size of the spheres is proportional to the size of the Martini 3 beads. (b) Constraints (black) and bonds (green) of the CG model and distribution of the partial charges at CG level. (c) Solvent accessible surface area (SASA) for the aromatic core of C8S3, atomistic (blue) and CG (red).

The first step of the CG model preparation was selecting a mapping for the aromatic core that would be rigid enough to maintain the flat surface of the benzimidazole rings and, at the same time, flexible enough to adopt different orientations of the side chains. The mapping of the cyanine aromatic core and C8S3 tails is presented in Fig. 3a. The benzimidazole rings are mapped with 4 tiny beads and 1 virtual site at their centre of geometry, following the suggested mapping of small ring-containing molecules with Martini 3.^45^ Additionally, there is a dummy particle (black sphere) that does not interact with other beads, but it allows the definition of the axis (dashed line) around which the benzimidazole ring is able to rotate. The substituents of the aromatic core are mostly represented by small beads, as commonly used for chemical fragments consisting of three heavy atoms or four heavy atoms in a branched configuration.^26^ There are also two tiny beads that connect the aromatic core with the aliphatic tails of C8S3. C8S3 has eight carbon atoms in each aliphatic tail that could be also mapped with two regular beads. We decided to use a mapping with tiny and small beads, in order to have a smoother transition to the aromatic core that is mapped mainly with tiny beads.

#### 2.1.2. Bead types

Martini 3 offers a wide variety of bead types covering a vast number of chemical groups.^26,45^ The selection of the bead types for the aromatic core of the cyanine dyes and each substituent is shown in Fig. 3a. Default Martini 3 bead types were used for the majority of the C8S3 chemical group, apart from the beads of the tails that are adjacent to the aromatic core. The main reason for this modification is the high polarisability of the aromatic core that should affect the atoms of the tails. Consequently, the first beads of the hydrophobic tails were changed by two interaction levels (TC2 was changed to TC4), and the beads of the hydrophilic tails were changed by four interaction levels (TC2 to TC6v) to capture the inductive effects from the SO3^−^ group. Finally, the aromatic core of these cyanine dyes is positively charged (+1) (see Fig. 3b). The extra charge was equally divided among the beads that include atoms of the polymethine chain (4xTN1q and TC4).

#### 2.1.3. Bonded distributions

Constraints hold together the aromatic rings, whereas (harmonic) bond potentials connect the polymethine bridge with the benzimidazoles (see Fig. 3b), and the aromatic core with the tails. There are also angles and dihedrals that control the orientation of the aromatic core. A detailed list of the bonded parameters is included in Fig. S2–S6 of the ESI.† The bonded distributions of the CG model were optimised to reproduce the atomistic distributions of the respective chemical groups. To obtain the atomistic distributions, a C8S3 monomer was solvated in a water box together with a counter ion and simulated for a short time. Then, a monomer of the CG model was simulated in the same way, and its bonded parameters were tuned by adapting its reference values and/or its force constants, until there was sufficient overlap. Most bonded distributions are well reproduced, because the current mapping favoured unimodal profiles. When bimodal distributions were encountered, capturing the mean values and the width of distributions was attempted. The comparison of the bonded distributions between the atomistic and the CG model are included in Fig. S2–S6 of the ESI.†

#### 2.1.4. Surface area

Maintaining the surface area of the atomistic models is extremely important in Martini 3, especially with the new small and tiny bead sizes that are able to describe aromatic rings with high detail. After building the initial CG models, the solvent accessible surface area (SASA) was calculated for the atomistic and the CG model (see Fig. 3c). More details regarding the SASA calculations and results are included in Fig. S7 of the ESI.†

### 2.2 Self-assembly of C8S3

Self-assembly simulations with the C8S3 CG model were performed in which randomly positioned molecules were solvated in cubic boxes filled with water and counter ions. The goal of these simulations was to study the early steps of nucleation, the morphology of the formed aggregates and the local arrangement of the C8S3 monomers, when small clusters are formed. Four systems were prepared with different numbers of molecules (50, 100, 500 and 1000 C8S3) and different box sizes. More details for each system are reported in Table S7 of the ESI.† All systems were analysed in terms of aggregate formation and local arrangement of the monomers with respect to each other.

**Fig. 4:**
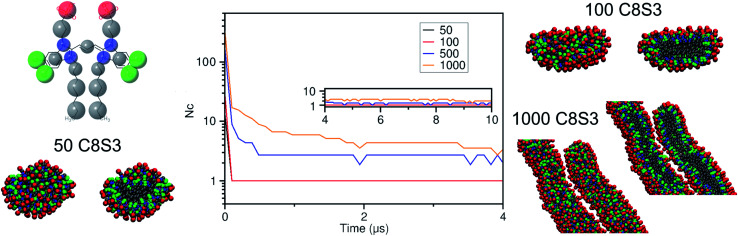
Quantification of the initial aggregation process of C8S3 molecules. Number of clusters (*N*_c_) formed as a function of time, for different system sizes containing 50, 100, 500, or 1000 monomers. The *y*-axis is plotted on a logarithmic scale. Note the data for the system size of 50 is largely underneath the red curve. On the left and right, snapshots from the last frame of different simulations are shown, together with the cross-sections of each aggregate. Their colouring scheme is based on the bead types of the C8S3 CG model (top left).

First, we calculated the number of clusters as a function of time, since this measurement gives an estimate of how fast the monomers aggregate, and whether the clusters merge to form larger structures. The number of clusters in each system was calculated by measuring the number of molecules within a certain cut-off distance. If the central beads of the aromatic core (TC4, Fig. 3a) of the monomers were within 1.5 nm, the molecules were accounted to be in the same cluster. In smaller systems, a cluster was rapidly formed and was maintained along the trajectory (see Fig. 4). A similar behaviour was also observed in the larger systems, but a single aggregate was not formed. Instead, the C8S3 molecules aggregated into several large clusters that did not merge during the simulated time scales. Both observations suggest that the hydrophobic effect is dominant in the beginning of the C8S3 self-assembly process, since the molecules immediately collapse into different structures, in which the alkyl tails are concealed from water. However, as the size of the aggregates increases, their fusion becomes slower. This behaviour could be attributed to the high energy barrier that prevents large aggregates from merging and keeping them kinetically trapped in worm-like structures.

It is known that the coarser representation in Martini or other CG methods smoothens the free energy landscapes of different processes and reduces the effective friction of the all atom representations.^46,47^ In general, a scaling factor of 2–10 applies when interpreting the time scales in simulations with the Martini force field.^48^ Regarding the C8S3 self-assembly, a comparison between simulated and actual time scales can only be done qualitatively to highlight the fast dynamics of C8S3 aggregation at the early nucleation steps. In reality, C8S3 self-assembly takes place in a few minutes, but such time scales are beyond the reach of the present molecular simulations techniques.

Snapshots from the last frames of each system show that the small systems formed small spherical blobs and the large systems formed elongated structures (Fig. 4). No aggregate formation similar to the tubular structures that are reported in the experiments was observed during our simulations. There are several reasons that could justify the different observations between the simulations and the experiments, such as the C8S3 concentration that was significantly larger in the simulations, the different timescales that diverge several orders of magnitude or the system sizes. These reasons are partially interconnected, and they highlight the complexity of simulating random self-assembly processes with either fine or coarse molecular models.

**Fig. 5:**
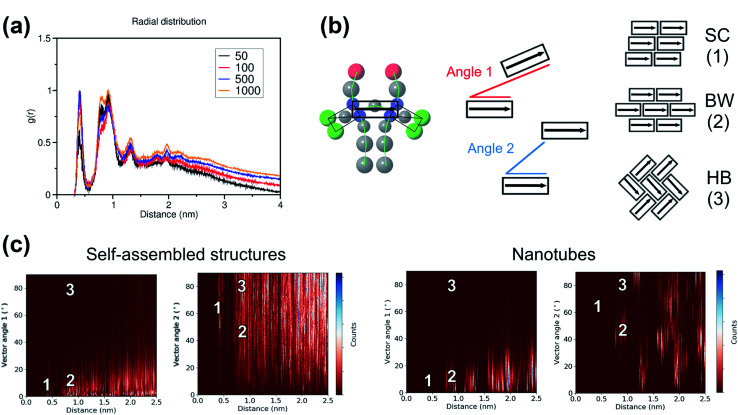
Local packing analyses of self-assembled and preformed aggregates. (a) RDF for the last 5 μs of each system. The profiles are calculated for the central bead of the aromatic core (TC4) and they are normalised based on the intensity of the highest peak in each profile. (b) Relative orientation of monomers. Each C8S3 molecule is represented by a sole vector that is used to describe the orientation of each monomer with respect to each other. Angle 1 represents the angle between the two vectors and angle 2 describes the angle between the head of a vector and the tails of the other vectors. Potential arrangements are shown as a graphical representation of stacked blocks: staircase (SC), brickwork (BW) and herringbone (HB). (c) Results from relative orientation analysis for a self-assembly (1000 C8S3 molecules, left) and a preformed nanotube simulation (right). The numbers highlight the location of the potential arrangements on the 2D-profiles.

To study the local arrangement inside the aggregates, the radial distribution function (RDF) of the central beads and the relative orientation of each monomer were measured at different time steps of the trajectories. Our results show that the local arrangement of the monomers is quite similar in all self-assembly simulations. Specifically, most of the molecules are located at a distance of either ∼0.4 nm or ∼0.8–0.9 nm (see Fig. 5a). The relative orientation of the C8S3 molecules was described as a function of distance and angles between each monomer (see Fig. 5b). To calculate the angles, the aromatic cores were represented as vectors defined by the position of the dummy particles (see Fig. 3a). Three different formations are used as reference: staircase (SC), brickwork (BW) and herringbone (HB). Molecule pairs at a distance of ∼0.8 nm could indicate either BW or HB arrangements, but the relative orientation analysis shows that the BW arrangement is preferred, since the angle between the vectors is ∼10° (see Fig. 5c). At ∼0.4 nm distance, the molecules arrange in a SC formation. SC arrangements are characteristic in self-assembled aggregates that present blue-shifted absorption spectra compared to the absorption of the solvated monomers.^49^ The C8S3 nanotubes present red-shifted absorption spectra indicating a BW or HB formation of the monomers in the final structures, but there is no evidence showing that C8S3 molecules do not undergo a wide search of possible conformations during their self-assembly.

The same analyses were performed at different time steps of the simulations and for every simulated system, but the results are roughly the same in all systems (see Fig. S8 and S9 of the ESI†). This indicates that, even though the initial aggregation of the C8S3 monomers is extremely fast, the preferred packing modes present large variety at the early stage of the self-assembly process. Large scale rearrangements and acquisition of discrete formations are likely to take place at longer time scales.

### 2.3 Preformed nanotubes

**Fig. 6:**
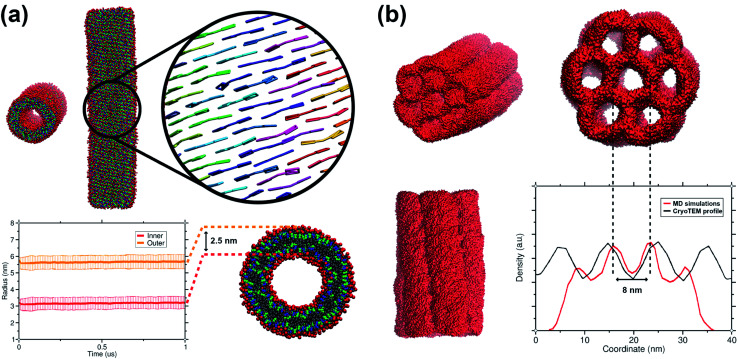
Structural features of C8S3 preformed structures. (a) Snapshots from a C8S3 nanotube simulation with the CG model (top panel). The arrangement of the C8S3 molecules is highlighted by representing the aromatic core of each monomer with different colours. By monitoring the position of the charged beads (SQ4*n*), we calculated the inner and outer wall radius of the nanotube along the trajectories (bottom panel). The nanotube thickness was calculated by the difference of these two values, and it was approximately 2.5 nm. (b) Snapshots of the C8S3 bundle simulation. The density profile from our simulations is superimposed to the experimental contrast profile from cryoTEM.^16^

The C8S3 CG model was also used for simulating preformed nanotubes of different size and measuring their structural features. All simulated nanotubes maintained their tubular structure along the trajectories (1 μs). A snapshot from the final step of a nanotube simulation is shown in Fig. 6a. The diameters of the designed systems ranged from 12 to 17 nm, close to the reported experimental values in the literature for the nanotube diameter that vary from 13 to 17 nm.^10,50^ Previous atomistic MD studies showed that preformed nanotubes with different diameter can be stable, as long as the nanotube thickness and the initial monomer arrangement is reasonable.^20,21^ In a previous study, we have shown that the thickness of the C8S3 nanotubes was consistently overestimated in the literature (3.5–4 nm), whereas the actual C8S3 nanotube thickness is ∼2.5 nm.^20^ All systems were constructed with an initial double-wall thickness of ∼2.5 nm, which remained roughly the same in all simulations. The values for the final dimensions of the stable simulated nanotubes are reported in Table S8 of the ESI.†

Among the potential arrangements, only the brickwork formation was stable with our CG model. Attempts with the herringbone formation resulted in deformed nanotubes during the beginning of the production phase (250–500 ns). A possible explanation is that the coarser representation of the C8S3 aromatic core favours stacked conformations. This behaviour can be seen in aromatic rings, especially as their size increases.^45^ Snapshots of a deformed nanotube are included in Fig. S10 of the ESI.† The staircase formation was not considered for C8S3 nanotube simulations, since it cannot reproduce the experimental spectra and it was proven to be unstable in atomistic simulations.^20^

Last but not least, we used the C8S3 CG model to simulate a C8S3 bundle, in other words, several nanotubes grouped together (see Fig. 6b). Bundles are the result of nanotube agglomeration, and they are formed when samples of C8S3 nanotubes are stored for several weeks at room temperature.^16,17^ The bundles can be either straight or twisted, but the driving forces of this morphological difference are unknown. Despite the small defects in the final structure, the overall shape of the bundle was maintained along the simulation. The final structure looks quite similar to the suggested model from Electron Microscopy experiments.^16^ In our case, the centre-to-centre distance is 8 nm, which is slightly smaller when compared to the experimental measured distance (10 ± 1 nm). A schematic representation of the C8S3 bundle construction is shown in Fig. S11 of the ESI.†

## 3 Conclusions

A combined top-down and bottom-up approach was followed to create a CG model for the C8S3 molecule, in line with the Martini 3 parametrization philosophy. Specifically, distributions from atomistic simulations and experimental partitioning free energies (among other target data) were used as reference for fine tuning the Martini 3 force field parameters. In the C8S3 CG model, QM calculations and atomistic simulations provided the bases for constructing and testing our model. During this procedure different aspects of the CG features were optimised to ensure that the final model accurately represents the structural and chemical properties of the cyanine dyes. Initially, QM methods were used to obtain optimal parameters for the description of the polymethine bridge dihedrals and different sets of partial charges. Then, the obtained parameters were tested on available crystal structures for cyanine dyes with the same aromatic core. The best set of parameters was used for atomistic simulations that provided the reference point for constructing the cyanine dye CG model. The CG model was further optimised to reproduce better the structural features of the monomer conformation when aggregates are formed. The final CG model represented with great detail structural/spatial properties of the cyanine dyes. Our results provide guidelines and best practices for constructing CG models for simple or complex nanomaterials.

Once the optimisation procedure was over, the C8S3 CG model was used in self-assembly simulations. Even though the assembled structures were not similar to the experimental determined structures, we observed different types of aggregates. Such a behaviour was expected, since the actual time for the C8S3 nanotube formation is in the time range of minutes. These time scales are beyond the accessible length of MD simulations, even for CG methods. However, the transition from amorphous blobs to elongated wires and the exploration of different arrangements during the early nucleation phase could be part of the mechanism of the C8S3 nanotube formation.

The performance of the CG model was tested by performing simulations of preformed C8S3 nanotubes. The tubular double-wall formation was maintained, suggesting that the CG model is compatible with the nanotube structure. However, our CG model favours brickwork-like formations, whereas computational modelling of the optical properties of the C8S3 nanotubes suggest that herringbone arrangements can describe the experimental optical signature better.^14,21^ Potential reasons for this preference could be some lack of interaction directionality due to coarse-graining or the absence of stronger partial charges that would make up for the high polarisability of the aromatic core. Further refinement of the aromatic core could also improve the self-assembly behaviour of C8S3 molecules.

As a proof of principle, a C8S3 bundle was designed and simulated with our CG model. The formation of bundles has been reported in experimental studies,^15,16^ but there is no literature on MD simulations of these systems. The size of the C8S3 bundles (>50 nm wide and >100 nm long) renders their simulation extremely computationally demanding, suggesting that coarser simulation methods might be more suitable for studying these systems.^51^ Despite its computational cost and limitations, we show that our protocol could be used to simulate such systems and calculate their structural features in more detail.

## Data availability

Structure and topology files can be downloaded from the Martini web-portal (https://cgmartini.nl).

## Conflicts of interest

There are no conflicts to declare.

## Supplementary Material

NA-004-D2NA00158F-s001
