# Supplementary material for: Modelling structural properties of cyanine dye nanotubes at coarse-grained level
Source: Nanoscale Adv. 2022 Jun 20;4(14):3033–42. doi: 10.1039/d2na00158f (PMC9419059; doi:10.1039/d2na00158f)
Supplement: NA-004-D2NA00158F-s001 [file NA-004-D2NA00158F-s001.pdf]

Electronic Supplementary Material (ESI) for Nanoscale Advances.
This journal is © The Royal Society of Chemistry 2022

Electronic Supplementary Information (ESI) for

Modelling structural properties of cyanine dye nanotubes at coarse-grained level

Ilias Patmanidis,[a,b] Paulo C. T. Souza,[c] Selim Sami,[a,b,d] Remco W. A. Havenith,[b,d,e] Alex H. de Vries,[a,b] Siewert J. Marrink*[a,b]

[a] *Groningen Biomolecular Science and Biotechnology Institute, University of Groningen, Nijenborgh 7, Groningen 9747 AG, the Netherlands.*
[b] *Zernike Institute for Advanced Materials, University of Groningen, Nijenborgh 4, Groningen 9747 AG, The Netherlands*
[c] *Molecular Microbiology and Structural Biochemistry, UMR 5086 CNRS and University of Lyon, Lyon, France.*
[d] *Stratingh Institute for Chemistry, University of Groningen, Nijenborgh 4, Groningen 9747 AG, The Netherlands*
[e] *Ghent Quantum Chemistry Group, Department of Chemistry, Ghent University, Krijgslaan 281 (S3) , B-9000 Gent, Belgium*

E-mail: s.j.marrink@rug.nl

1 Parameter optimisation

The bonded terms for the polymethine bridge and the partial charges of the aromatic core (C0C0) were optimised based on QM calculations. Specifically, the torsional potentials to be added to the dihedral between atoms C2-C3-C5-N9 and C4-C2-C3-C5 have been determined based on the energy difference between the QM and the MD dihedral profiles. For this purpose, models for the C0C0 (Figure 1b of the main text, where R represents hydrogen atoms) and C1C1 (Figure 1b, where R represents methyl groups) molecules were used. The atoms of these aromatic cores are the same as the C8S3 core, but the simple side chains allow us to focus solely on the polymethine bridge and the conjugated aromatic core. QM dihedral profiles were obtained by performing a relaxed scan with angle increments of 5 degrees using the Gaussian16 software[?] . For these scans, ωB97xD functional with 6-311G(d,p) basis set was used. MD dihedral profiles were obtained by performing similar relaxed dihedral scans, where the existing dihedral potentials on the dihedrals of interest were removed. Then, the difference between the QM and MD profiles was fitted to a Ryckaert-Bellemans (RB) type of function, Eq. **??**. The point charges for each system were generated after optimising the C0C0 and C1C1 structures with the Hartree-Fock (HF) method and 6-31G* basis set in two different ways: Dipole Preserving Analysis (DPA)[?] using the GAMESS-UK software[?] , and Restricted Electrostatic Potential (RESP)[?] method using the Gaussian16 software[?] . The force constants for optimised MD parameters are reported in Table **??**.

$$V_{RB} = \sum_{n=0}^{5} C_n(\cos(\theta - 180))^n \tag{1}$$

n is the number of added potentials, C_n is the RB coefficient for each potential and θ is the dihedral angle.

Molecule	Method	Dihedral	C1 (kJ/mol)	C2 (kJ/mol)	C3 (kJ/mol)	C4 (kJ/mol)	C5 (kJ/mol)	C6 (kJ/mol)
C0C0	HF-DPA	C5-C3-C2-C4	103.0725	35.0723	-131.2985	-26.7065	48.1962	10.6666
		N9-C5-C3-C2	57.7083	5.2693	-79.2583	-9.3074	23.1247	5.1088
C0C0	HF-RESP	C5-C3-C2-C4	96.3923	23.1281	-134.1660	-28.4803	47.4333	13.6272
		N9-C5-C3-C2	62.1772	1.8762	-89.1708	-3.8494	27.5176	2.0697
C1C1	HF-RESP	C5-C3-C2-C4	99.6354	26.5038	-136.8010	-37.0012	52.2696	22.8211
		N9-C5-C3-C2	59.4377	4.6123	-85.0340	-11.1293	27.2898	6.3321

Table S1: Force constant coefficients for RB potential functions for C0C0 and C1C1 with different methods.

It is evident that the choice of QM method affects the results and the values for the force constant coefficients show variations. However, the values are not dramatically different. Even for C0C0 and C1C1, the force constant coefficients are pretty similar. The R squared for all fitted profiles was ∼0.99, suggesting the MD profiles are almost identical to the QM ones. An overlap of the energy profiles between the QM and MD methods for C0C0 and C1C1 is shown in Figure **??**. Since the values for the RB coefficients of the dihedral angles are similar between C0C0 and C1C1, we can assume that the contribution of the substituents does not significantly affect the potentials for the rotation around the bonds of the polymethine

bridge. To maintain simplicity, the respective C0C0 force constant coefficients were used for the simulated cyanine dyes.

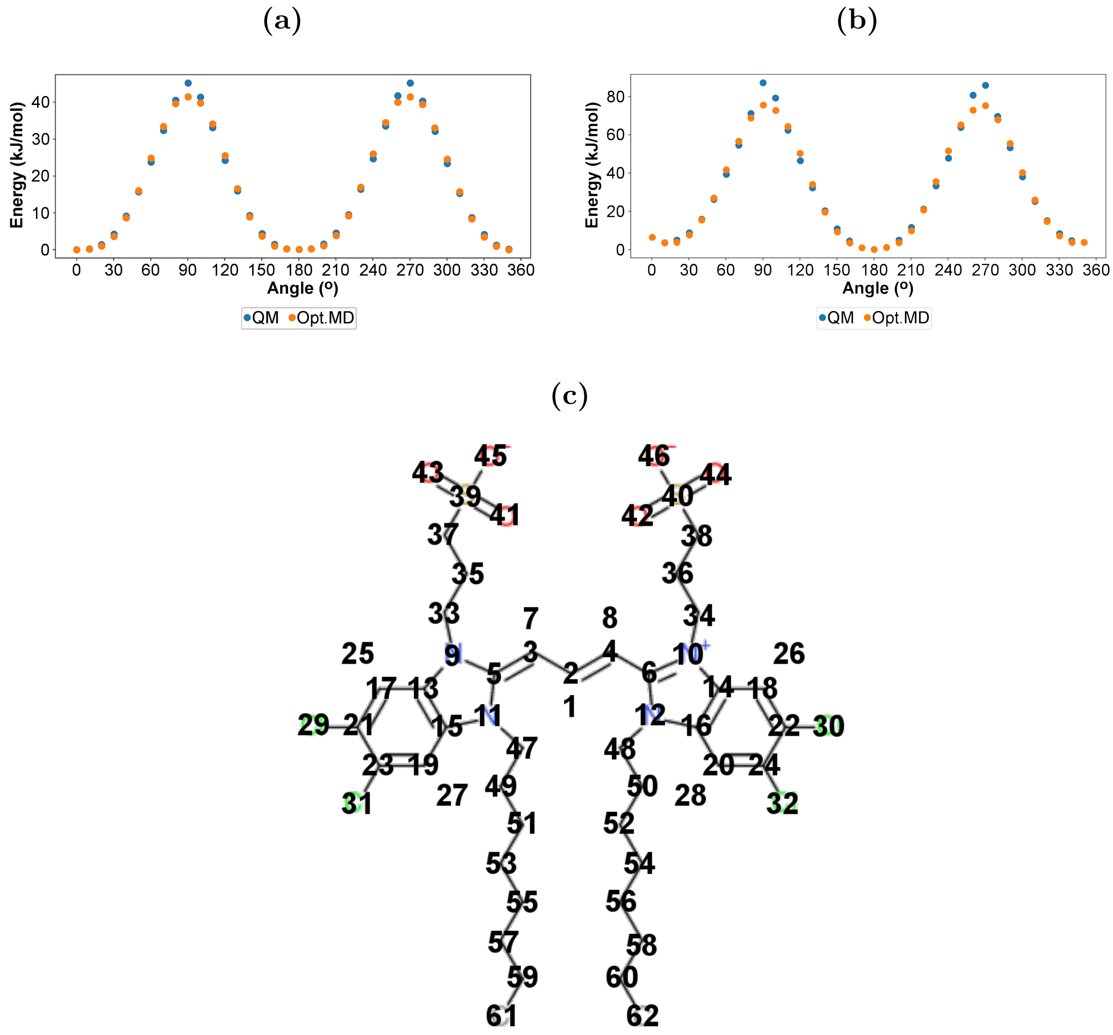

Figure S1: Potential energy profiles for dihedrals of C0C0 for the HF-RESP method. a) C5-C3-C2-C4. b) N9-C5-C3-C2. c) Atom index of C8S3 atomistic model. The atom numbers of the aromatic core among the generated models are identical.

After obtaining an optimal set of parameters for the dihedrals of the polymethine bridge at QM level, we compared different methods for calculating partial charges based on their ability to reproduce structural features from experimental data. Specifically, atomistic simulations of the crystal structures of two cyanine dye, C2C2[?] and C8O3[?], were used to evaluate the performance of three different models:

- Model 1: DPA charges from QM calculations (B3LYP/6-31G*) on C1C1 and cosinoid dihedral definitions[?]

- Model 2: DPA charges (HF/6-31G*) from QM calculations (ωB97xD/6-311G**) and RB dihedral definitions
- Model 3: RESP charges (HF/6-31G*) from QM calculations (ωB97xD/6-311G**) and RB dihedral definitions

The partial charges for each model of C2C2 and C8O3 are reported in Tables ??-??.

Atom	Model 1	Model 2	Model 3
H1	0.030	0.133	0.224
C2	-0.030	-0.017	0.148
C3	-0.250	-0.448	-0.606
C4	-0.250	-0.448	-0.606
C5	0.660	0.852	0.406
C6	0.660	0.852	0.406
H7	0.030	0.116	0.248
H8	0.030	0.116	0.248
N9	-0.390	-0.539	-0.169
N10	-0.390	-0.539	-0.169
N11	-0.390	-0.539	-0.169
N12	-0.390	-0.539	-0.169
C13	0.280	0.334	0.094
C14	0.280	0.334	0.094
C15	0.280	0.334	0.094
C16	0.280	0.334	0.094
C17	-0.160	-0.227	-0.220
C18	-0.160	-0.227	-0.220
C19	-0.160	-0.227	-0.220
C20	-0.160	-0.227	-0.220
C21	0.020	0.014	0.072
C22	0.020	0.014	0.072
C23	0.020	0.014	0.072
C24	0.020	0.014	0.072
H25	0.110	0.170	0.191
H26	0.110	0.170	0.191
H27	0.110	0.170	0.191
H28	0.110	0.170	0.191

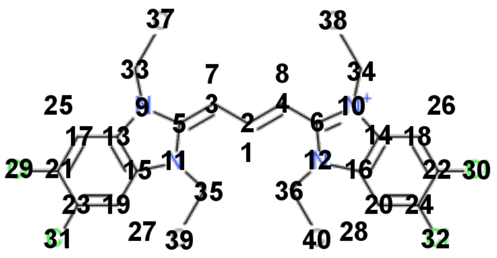

Atom	Model 1	Model 2	Model 3
CL29	-0.030	-0.055	-0.065
CL30	-0.030	-0.055	-0.065
CL31	-0.030	-0.055	-0.065
CL32	-0.030	-0.055	-0.065
C33	0.200	0.264	0.206
C34	0.200	0.264	0.206
C35	0.200	0.264	0.206
C36	0.200	0.264	0.206
C37	0.0	0.0	0.024
C38	0.0	0.0	0.024
C39	0.0	0.0	0.024
C40	0.0	0.0	0.024

Table S2: Partial charges for different C2C2 models.

The structural features of the crystal structures that were evaluated were the dimensions of the supercell (simulation box) and its density. Additionally, the root-mean-square deviation (RMSD) from the initial conformation and the position of the first peak in Radial Distribution Function (RDF) calculations are reported. The results from the crystal simulations are reported in Table ??.

In general, even though the differences are quite small, the models with HF-RESP charges performed better in terms of maintaining the structural properties of the cyanine crystal structures. Model 1 performed worse than the others, especially in the C8O3 crystal. The

results for Model 2 and Model 3 are quite similar, but Model 3 reproduced slightly better the position of the first peak in the RDF calculation. The parameters of Model 3 were used for the atomistic simulations of the C8S3 monomer in water. The partial charges for C8S3 are reported in Table **??**.

Atom	Model 1	Model 2	Model 3
H1	0.030	0.166	0.21
C2	-0.030	-0.006	0.133
C3	-0.250	-0.499	-0.562
C4	-0.250	-0.499	-0.562
C5	0.660	0.902	0.420
C6	0.660	0.902	0.420
H7	0.030	0.164	0.282
H8	0.030	0.164	0.282
N9	-0.390	-0.565	-0.185
N10	-0.390	-0.565	-0.185
N11	-0.390	-0.565	-0.185
N12	-0.390	-0.565	-0.185
C13	0.280	0.338	0.112
C14	0.280	0.338	0.112
C15	0.280	0.338	0.112
C16	0.280	0.338	0.112
C17	-0.160	-0.224	-0.234
C18	-0.160	-0.224	-0.234
C19	-0.160	-0.224	-0.234
C20	-0.160	-0.224	-0.234
C21	0.020	0.011	0.067
C22	0.020	0.011	0.067
C23	0.020	0.011	0.067
C24	0.020	0.011	0.067
H25	0.110	0.159	0.188
H26	0.110	0.159	0.188
H27	0.110	0.159	0.188
H28	0.110	0.159	0.188
CL29	-0.030	-0.085	-0.094
CL30	-0.030	-0.085	-0.094
CL31	-0.030	-0.085	-0.094
CL32	-0.030	-0.085	-0.094
C33	0.200	0.215	0.120
C34	0.200	0.215	0.120
C35	0.0	0.069	0.0
C36	0.0	0.022	0.044
C37	-0.080	-0.062	0.019
C38	0.120	-0.124	-0.093
C39	0.640	0.918	0.865
C40	0.600	0.877	0.878

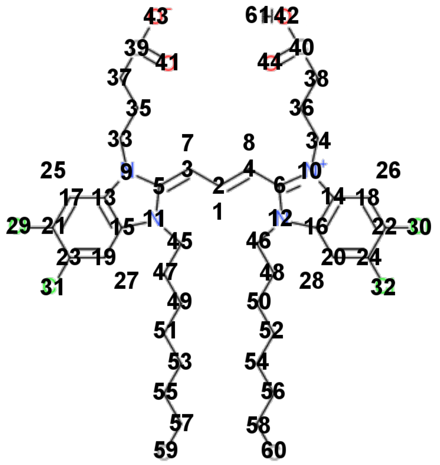

Atom	Model 1	Model 2	Model 3
O41	-0.780	-0.722	-0.782
O42	-0.610	-0.784	-0.682
O43	-0.780	-0.722	-0.782
O44	-0.570	-0.685	-0.651
C45	0.200	0.192	0.120
C46	0.200	0.192	0.120
C47	0.0	0.031	0.089
C48	0.0	0.031	0.089
C49	0.0	-0.007	-0.001
C50	0.0	-0.007	-0.001
C51	0.0	0.017	0.035
C52	0.0	0.017	0.035
C53	0.0	0.0	-0.009
C54	0.0	0.01	-0.009
C55	0.0	0.016	-0.002
C56	0.0	0.016	-0.002
C57	0.0	0.05	0.062
C58	0.0	0.05	0.062
C59	0.0	-0.05	-0.068
C60	0.0	-0.05	-0.068
H61	0.460	0.455	0.453

Table S3: Partial charges for different C8O3 models.

			Model 1		Model 2		Model 3	
Molecule	Analysis	Reference	Average	Error	Average	Error	Average	Error
C2C2	X (nm)	4.012	4.218	0.001	3.989	0.001	4.003	0.003
	Y (nm)	3.310	3.444	0.001	3.480	0.001	3.426	0.001
	Z (nm)	6.451	6.015	0.001	6.127	0.001	6.160	0.001
	Density (kg/m^3)	1442	1470.09	0.17	1510.11	0.41	1520.69	0.21
	RMSD (nm)		0.311		0.187		0.118	
	RDF (nm)	0.868	0.807		0.883		0.875	
C8O3	X (nm)	4.974	5.326	0.004	4.925	0.001	4.999	0.006
	Y (nm)	4.682	5.184	0.008	4.765	0.001	4.695	0.003
	Z (nm)	5.201	4.422	0.005	5.131	0.002	5.141	0.008
	Density (kg/m^3)	1254	1245.19	0.56	1262.47	0.23	1260.11	0.50
	RMSD (nm)		1.081		0.877		0.885	
	RDF (nm)	0.420	0.487		0.453		0.418	

Table S4: Summary of crystal simulations analysis. Different structural features of the simulated systems are compared with the initial values of the crystal structure? ? . The RMSD is calculated for every atom of the cyanine dyes. The RDF value refers to the position of the first peak for the distribution of the central atom of the cyanine core (C2). The error is calculated based on block averages over 5 blocks. The cells of the table are coloured based on the value of the simulated feature compared to the initial value. Green colour indicates that the reported value is ±1-2% of the initial value. Yellow represents ±2-5%, orange ±5-10% and red >10%.

Atom	Model 3	Atom	Model 3	Atom	Model 3	Atom	Model 3
H1	0.020	C17	-0.205	C33	0.118	C49	0.050
C2	0.462	C18	-0.205	C34	0.118	C50	0.050
C3	-0.691	C19	-0.205	C35	0.099	C51	-0.045
C4	-0.691	C20	-0.205	C36	0.099	C52	-0.045
C5	0.538	C21	0.067	C37	-0.065	C53	-0.087
C6	0.538	C22	0.067	C38	-0.065	C54	-0.087
H7	0.246	C23	0.067	S39	1.324	C55	0.106
H8	0.246	C24	0.067	S40	1.324	C56	0.106
N9	-0.221	H25	0.189	O41	-0.706	C57	-0.026
N10	-0.221	H26	0.189	O42	-0.706	C58	-0.026
N11	-0.221	H27	0.189	O43	-0.706	C59	0.074
N12	-0.221	H28	0.189	O44	-0.706	C60	0.074
C13	0.100	CL29	-0.109	O45	-0.706	C61	-0.089
C14	0.100	CL30	-0.109	O46	-0.706	C62	-0.089
C15	0.100	CL31	-0.109	C47	0.183		
C16	0.100	CL32	-0.109	C48	0.183		

Table S5: Partial charges used for the C8S3 molecule.

2 C8S3 nanotube structures

System	No C8S3	Inner radius PMB (nm)	Inner rolling angle (°)	Outer radius PMB (nm)	Outer rolling angle (°)	Simulation time (ns)	Length (nm)
1	3626	3.72	30.96	5.49	31.53	1000	50
2	3795	3.96	42.11	5.59	41.99	1000	50
3	4350	4.90	41.87	6.64	42.88	1000	50
4	4883	5.31	32.73	7.08	32.73	1000	50
5	6071	6.84	36.01	8.57	32.42	1000	50
6H	3626	3.72	30.96	5.49	31.53	500	50
7B	16042	3.72	30.96	5.49	31.53	1000	50

Table S6: Initial parameters for the preparation of C8S3 nanotubes simulations. H stands for herringbone and B stands for bundle.

3 C8S3 coarse-grained model

In Martini 3, the mapping of aromatic moieties is based on the centre of geometry of all atoms (heavy and hydrogen atoms) that participate in each chemical group. However, this rule is slightly flexible, since parametrization aims at optimising the surface area of the molecules, and different definitions can be used to maintain the overall shape. In the C8S3 model, constraints hold together the aromatic rings, whereas bonds connect the polymethine bridge with the benzimidazoles, Figures S2 and S4. Each benzimidazole ring is constituted by four normal beads, one virtual site at their centre of geometry and one dummy particle. Virtual sites have no mass, so the mass of the respective bead has been evenly distributed to the four normal beads. In contrast to virtual sites, dummy particles do not interact via non-bonded interactions with any bead, and only act as supportive particles to allow specific conformations. The angles and dihedrals that control the orientation of the aromatic core are presented in Figures S3, S5 and S6. There were no angles between the side chains and the central bead to allow free rotation around the defined axis. No dihedral definitions were used between the core and side chains, since the angle definitions were sufficient to describe the movement of the side chains. Finally, the aromatic core of these cyanine dyes is positively charged. The extra charge was assigned to the beads that represent atoms of the polymethine chain.

The solvent accessible surface area (SASA) was calculated for the atomistic and the CG model, in order to compare the final size of the models. The initial mapping underestimated the volume of the aromatic core. Overlapping the volume of the atomistic and CG models revealed that the area around the chlorines was slightly smaller in the initial CG models (Model 1). Consequently, the mapping was modified to increase the overlap of the two surfaces. Instead of mapping the SX3 bead on the centre of geometry of the CHCCl group (Model 1), the bead was placed at the centre of the C-Cl bond (Model 2). The surface of the aromatic core with the new mapping is almost identical to the atomistic value, Figure **??**b.

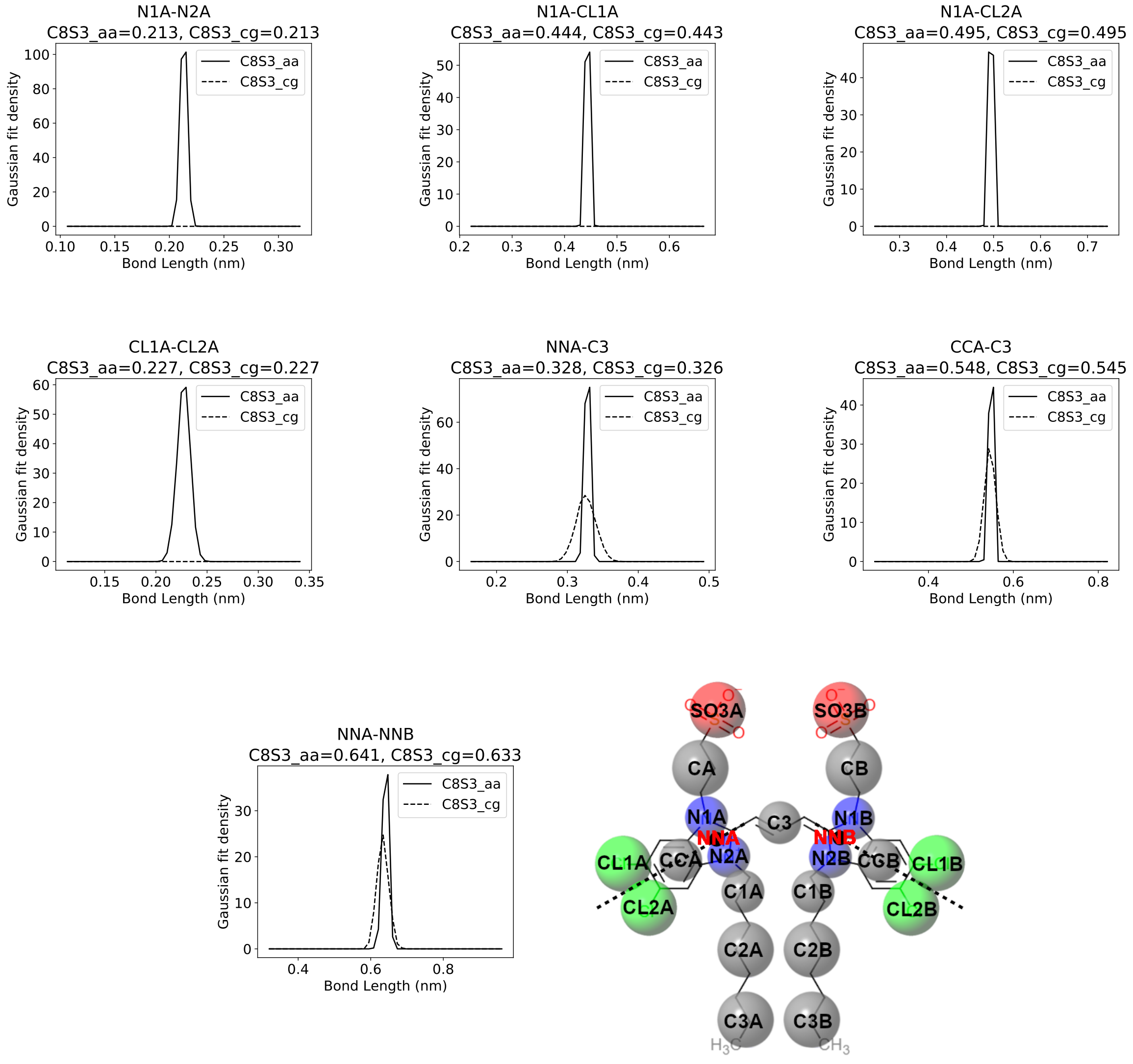

Figure S2: Comparison of atomistic and CG bond length distributions for the aromatic core of C8S3.

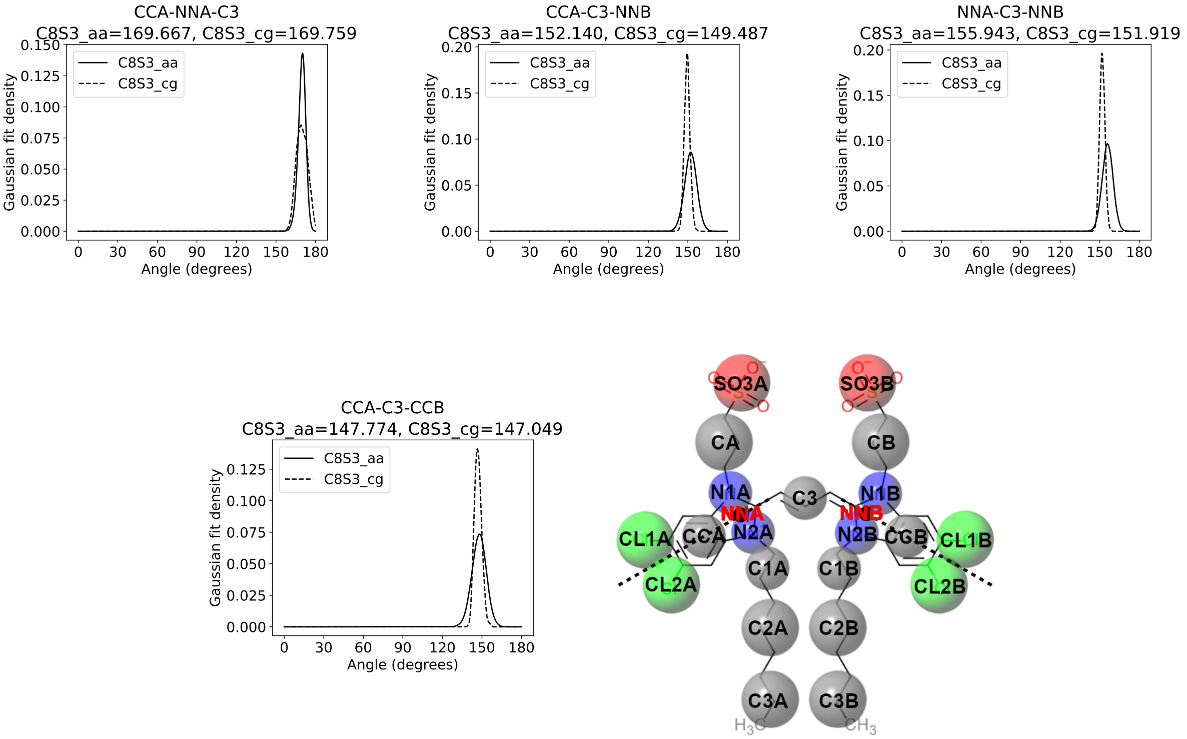

Figure S3: Comparison of atomistic and CG angle distributions for the aromatic core of C8S3.

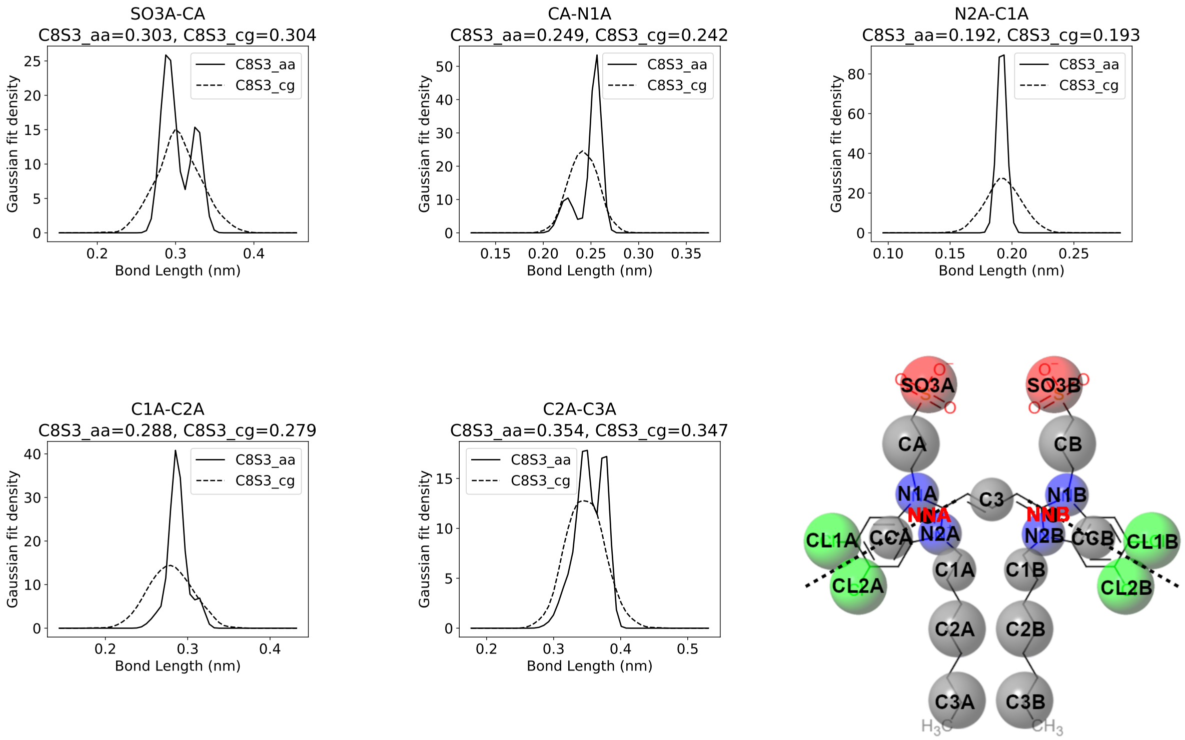

Figure S4: Comparison of atomistic and CG bond length distributions for the tails of C8S3.

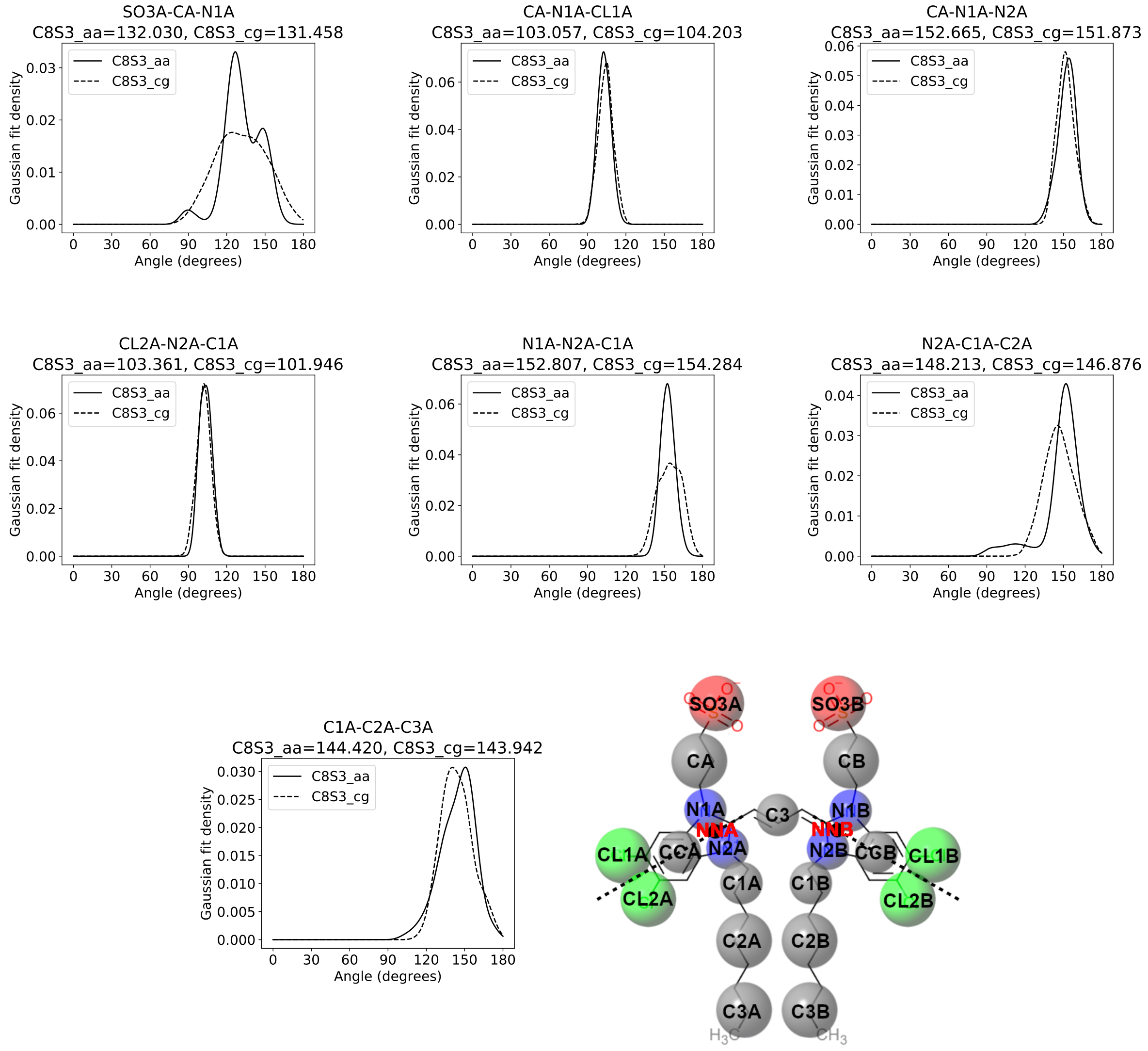

Figure S5: Comparison of atomistic and CG angle distributions for the tails of C8S3.

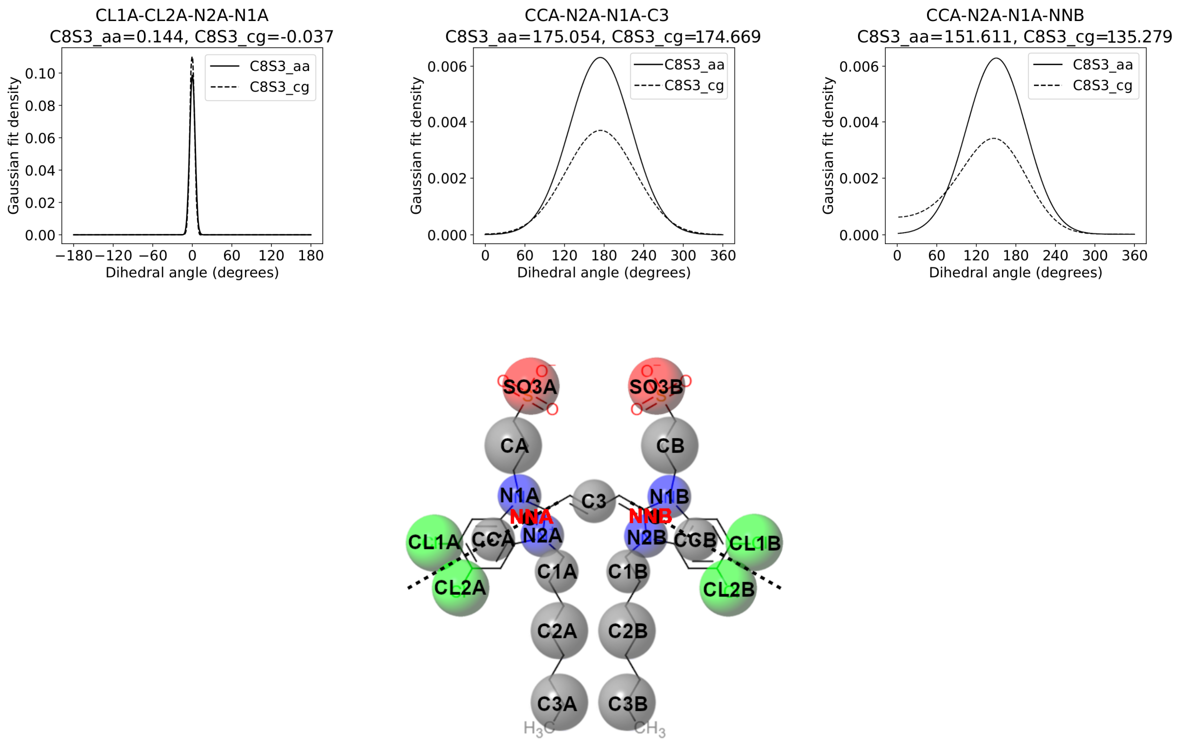

Figure S6: Comparison of atomistic and CG dihedral distributions for C2C2.

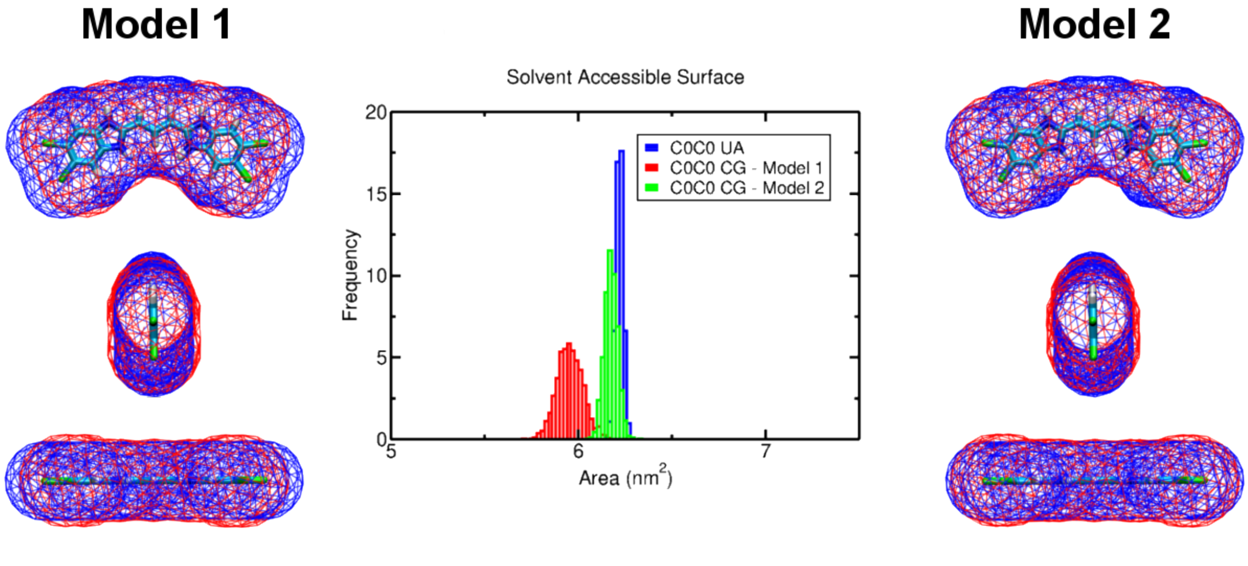

Figure S7: Solvent accessible surface area for the aromatic core of the C8S3 molecule. The histograms represent the SASA for one monomer in solution for a trajectory of 100 ns. The number of dots for SASA in the histogram analysis was set to 10000, whereas for the surface representation the number of dots were 50.

4 Self-assembly of C8S3

System	No C8S3	No Na	No W	Box size (nm)	Simulation time (µs)
1	50	50	7329	10x10x10	10
2	100	100	6726	10x10x10	10
3	500	500	58309	20x20x20	10
4	1000	1000	114828	25x25x25	10

Table S7: System details for random self-assembly simulations.

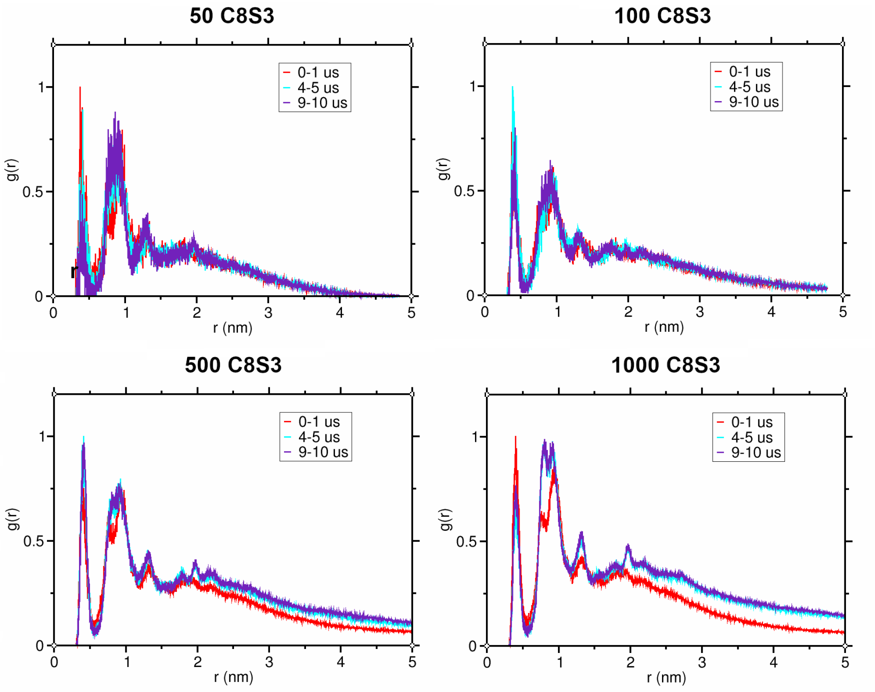

Figure S8: RDF calculations for different time frames of each simulated system.

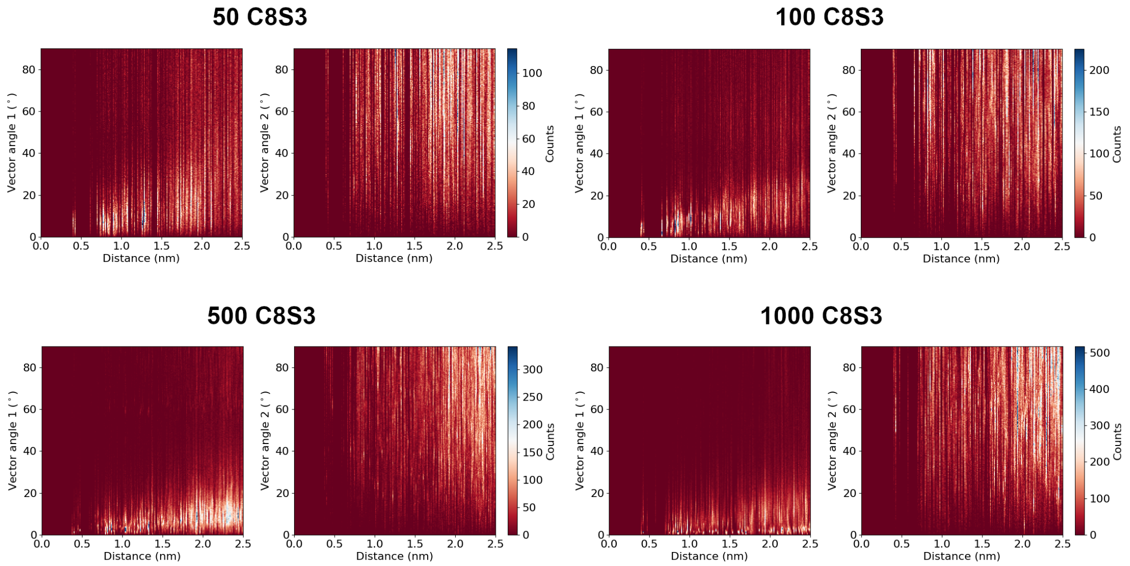

Figure S9: Relative orientation analysis for each simulated system.

5 Simulations of preformed C8S3 structures

System	Inner radius (nm)	Outer radius (nm)	Thickness (nm)
1	3.07±0.36	5.52±0.31	2.47±0.07
2	3.40±0.39	5.85±0.36	2.47±0.15
3	4.01±0.41	6.47±0.38	2.48±0.04
4	4.57±0.39	7.05±0.35	2.48±0.05
5	6.02±0.41	8.51±0.38	2.49±0.08

Table S8: Final dimensions of C8S3 nanotube simulations (mean values and standard deviations).

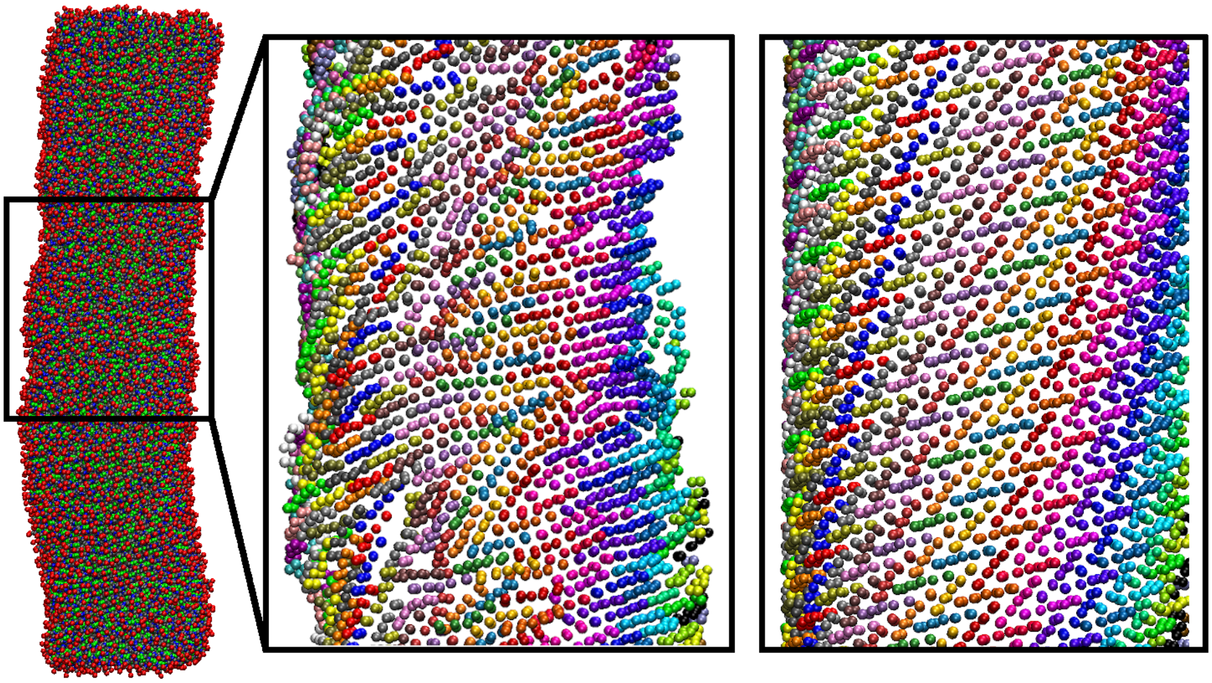

Figure S10: Snapshots from a C8S3 nanotube simulation with the herringbone arrangement after 500 ns in the production phase (left panel). Initial arrangement of C8S3 molecules (right panel).

6 C8S3 bundle preparation

The C8S3 bundle construction can be summarised in following steps: i) preparation of a single C8S3 nanotube (System 1), ii) replication of the single nanotube and translation of the nanotubes' position until the optimal thickness (2.5 nm) is achieved, and iii) removal of the overlapping C8S3 molecules of the outer wall.

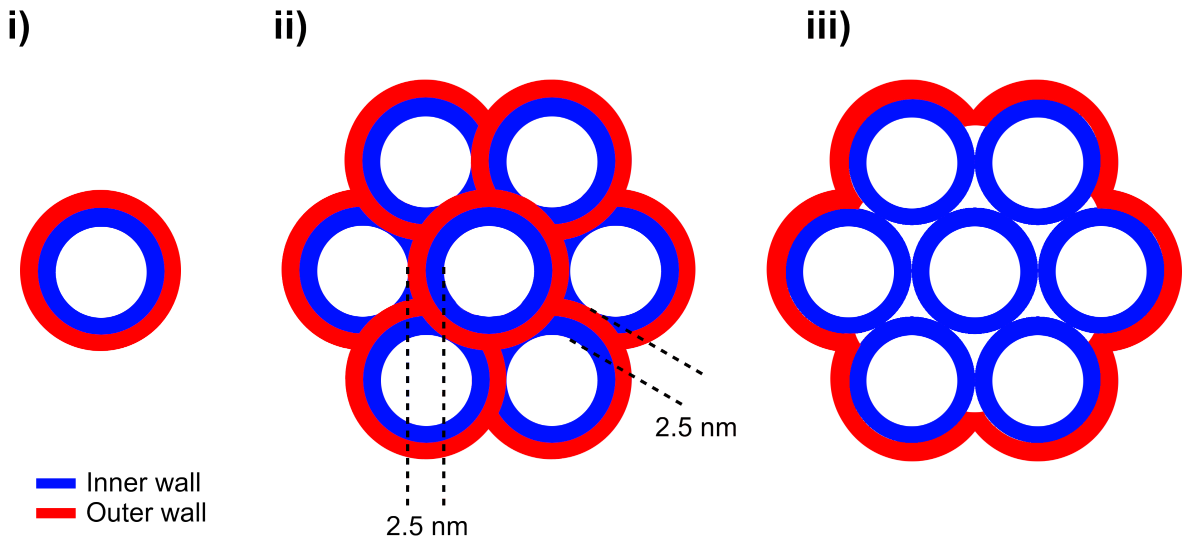

Figure S11: Schematic representation of the C8S3 bundle preparation.

References

[] M. J. Frisch, G. W. Trucks, H. B. Schlegel, G. E. Scuseria, M. A. Robb, J. R. Cheeseman, G. Scalmani, V. Barone, G. A. Petersson, H. Nakatsuji, X. Li, M. Caricato, A. V. Marenich, J. Bloino, B. G. Janesko, R. Gomperts, B. Mennucci, H. P. Hratchian, J. V. Ortiz, A. F. Izmaylov, J. L. Sonnenberg, D. Williams-Young, F. Ding, F. Lipparini, F. Egidi, J. Goings, B. Peng, A. Petrone, T. Henderson, D. Ranasinghe, V. G. Zakrzewski, J. Gao, N. Rega, G. Zheng, W. Liang, M. Hada, M. Ehara, K. Toyota, R. Fukuda, J. Hasegawa, M. Ishida, T. Nakajima, Y. Honda, O. Kitao, H. Nakai, T. Vreven, K. Throssell, J. A. Montgomery, Jr., J. E. Peralta, F. Ogliaro, M. J. Bearpark, J. J. Heyd, E. N. Brothers, K. N. Kudin, V. N. Staroverov, T. A. Keith, R. Kobayashi, J. Normand, K. Raghavachari, A. P. Rendell, J. C. Burant, S. S. Iyengar, J. Tomasi, M. Cossi, J. M. Millam, M. Klene, C. Adamo, R. Cammi, J. W. Ochterski, R. L. Martin, K. Morokuma, O. Farkas, J. B. Foresman and D. J. Fox, *Gaussian˜16 Revision A.03*, 2016, Gaussian Inc. Wallingford CT.

[] B. T. Thole and P. T. van Duijnen, *Theoretica chimica acta*, 1983, **63**, 209–221.

[] M. F. Guest*, I. J. Bush, H. J. J. Van Dam, P. Sherwood, J. M. H. Thomas, J. H. Van Lenthe, R. W. A. Havenith and J. Kendrick, *Molecular Physics*, 2005, **103**, 719–747.

[] C. I. Bayly, P. Cieplak, W. Cornell and P. A. Kollman, *The Journal of Chemical Physics*, 1993, **97**, 10269–10280.

[] D. L. Smith and H. R. Luss, *Acta Crystallographica B*, 1972, **28**, 2793–2806.

[] S. Kirstein, H. von Berlepsch, C. Böttcher, C. Burger, A. Ouart, G. Reck and S. Dähne, *ChemPhysChem*, 2000, **1**, 146–150.

[] I. Patmanidis, A. H. de Vries, T. A. Wassenaar, W. Wang, G. Portale and S. J. Marrink, *Phys. Chem. Chem. Phys.*, 2020, **22**, 21083–21093.
